# Preparation of bismuth nanowire encased in quartz template for Hall measurements using focused ion beam processing

**DOI:** 10.1186/1556-276X-7-505

**Published:** 2012-09-07

**Authors:** Masayuki Murata, Yasuhiro Hasegawa, Takashi Komine, Tomohiro Kobayashi

**Affiliations:** 1 Saitama University, 255 Shimo-Okubo, Sakura-ku, Saitama, 338-8570, Japan; 2 Ibaraki University, 4-12-1 Nakanarusawa, Hitachi, Ibaraki, 316-8511, Japan; 3 RIKEN, 2-1 Hirosawa, Wako, Saitama, 351-0198, Japan; 4 Japan Society for the Promotion of Science (JSPS), Sumitomo-Ichibancho FS Bldg., 8 Ichibancho, Chiyoda-ku, Tokyo, 102-8472, Japan

**Keywords:** Bismuth nanowire, Hall measurement, focused ion beam, thermoelectrics, 81.07.Gf

## Abstract

Forming electrodes on opposite sides of an individual bismuth nanowire was attempted to prepare for Hall measurements. Although a 1-mm-long bismuth nanowire which is completely covered with a quartz template has been successfully fabricated to prevent oxidation, it is very difficult to attach Hall electrodes on the opposite sides of the nanowire due to the quartz covering. One side of the cylindrical quartz template was removed by polishing without exposure of the nanowire to the atmosphere; the thickness between the polished template surface and the nanowire was estimated to be several micrometers. Focused ion beam processing was successfully employed to expose both surfaces of the nanowire under high vacuum by removing part of the quartz template. A carbon thin film was then deposited *in situ* on the wire surface to fabricate an electrical contact on the bismuth nanowire sample. Furthermore, the energy dispersive X-ray analysis was performed to the area processed by focused ion beam, and the bismuth component of the nanowire was successfully detected. It was confirmed that the focused ion beam processing was applicable to attach electrodes to bismuth nanowire for Hall measurement.

## Background

Nanoscale structures such as superlattices and nanowires attract research interest due to their electrical transport properties. It has been expected that nanostructured thermoelectric materials would exhibit enhanced performance [1-5]. In particular, one-dimensional bismuth nanowires have been expected to show an enhanced figure of merit as thermoelectric materials [2,3]. Bismuth, as a semimetal, has interesting electrical properties such as small effective mass, low carrier density, and a long mean free path; and the properties of bismuth, such as its Fermi surface and effective mass, have been well studied [6,7]. Bismuth nanowires have been fabricated using several methods for the study of the thermoelectric properties of one-dimensional systems [8-13]. Our group has fabricated a bismuth microwire array using a glass template and individual nanowires using a quartz template by application of a liquid-phase high pressure injection method [14-24]. The quartz template possesses an individual hole of several-hundred-nanometer diameter and over 1 mm long and is fabricated by identical procedure of an optical fiber for us to make the nanowire [21]. The simultaneous measurement of the Seebeck coefficient and resistivity was successfully achieved using the bismuth nanowire. The results implied that the carrier mobility of a nanowire less than 1 μm in diameter was significantly reduced compared with that for a bulk bismuth sample due to the collision at the quartz template surface, which functioned as a boundary condition, as a result of the classical size effect [25,26]. Although we have considered variations in the mobility of each carrier in the nanowire sample using a mean free path limitation model, direct measurement results have not yet been reported. Discussions have been based only on the model and temperature dependence measurement results of Seebeck coefficient and resistivity. Therefore, the carrier density and mobility should be evaluated by Hall measurement.

Bismuth nanowires are usually fabricated by using alumina templates [8-11]. Since bismuth nanowires are covered with the alumina template, the template must be removed so that the electrodes can be attached at the opposite sides of the nanowire for Hall measurements. Even if the alumina template component was completely removed using acid, it has been reported that the bare surface of the bismuth nanowire is likely to be oxidized in the atmosphere. Therefore, removal of the oxide layer covering the nanowire surface is a very important process to achieve ohmic contact with the electrodes [27-29]. Recently, a new fabrication process for bismuth nanowires called on-film formation of nanowires was reported [30], where the oxidized surface was removed by ion beam sputtering, and electrodes were deposited *in situ* on the bismuth nanowire without breaking the vacuum. However, a majority of the bismuth nanowire area was oxidized.

In the current research, however, the bismuth nanowires were covered with a quartz template; therefore, oxidation of the wire surface is prevented. Although such nanowire has apparent advantages, it was not considered to be suitable for the four-wire and Hall measurements because it is very difficult to remove the quartz component locally. However, in the previous study, we had successfully fabricated electrodes on the surface of the bismuth nanowire with ohmic contact using polishing and focused ion beam (FIB) processing and performed four-wire resistance measurement [31]. A local area of the bismuth wire was successfully exposed, and a carbon electrode was deposited on the bismuth wire *in situ*. In this paper, this method was applied for the preparation of a Hall measurement sample and discussed for a nanowire covered with a quartz template. The quartz template was cut using FIB processing to successfully expose the opposite side surfaces of a bismuth nanowire under high vacuum, and a carbon electrode was then immediately deposited on the surface of the bismuth nanowire.

## Methods

**Figure 1:**
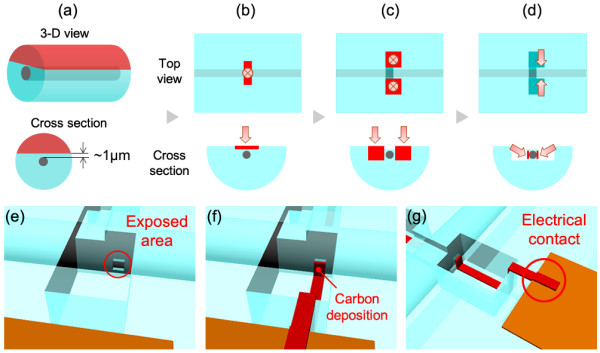
**Schematic diagram showing the processing for preparation of a bismuth nanowire for Hall measurements.** (**a**) Polishing, (**b**) detection of the nanowire location, (**c**) removal of the side parts of the template, (**d**) exposure of the wire surface, (**e**) 3-D view of the exposed wire surface, (**f**) carbon deposition to form electrical contacts, and (**g**) contact between carbon film and copper electrodes located on the top of the quartz.

**Figure 2:**
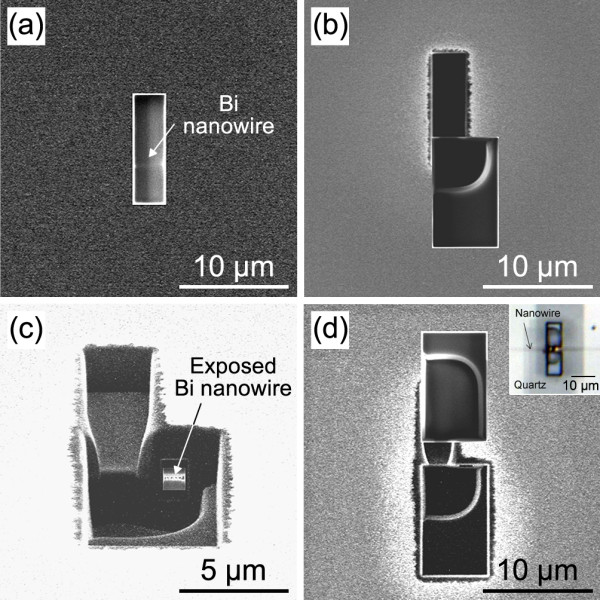
**SIM images during the FIB process.** (**a**) Detection of the nanowire location, (**b**) removal of the side parts of the template, (**c**) side view tilted at 60º to expose the nanowire surface, and (**d**) top view of the processed area after FIB processing. Inset of (**d**) shows optical microscope image of the processed area.

**Figure 3:**
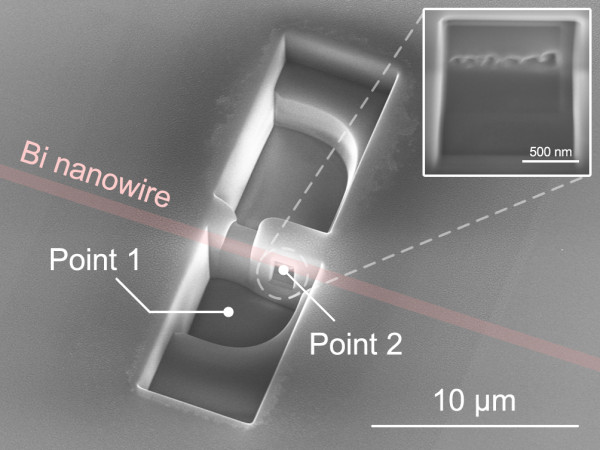
**SEM image of the FIB processed area.** The thick red line represents the location of the bismuth nanowire in the quartz template. The inset shows a magnified figure of the exposed side area of the nanowire. Points 1 and 2 indicate the locations used for EDX analysis.

An individual bismuth nanowire sample covered with a quartz template was prepared for the experiment. The diameter and length of the nanowire were *ca*. 700 nm and >1 mm, respectively. The individual nanowire was located in the center of the quartz template, of which the diameter was *ca*. 0.5 mm. The nanowire could be observed through the template using an optical microscope, due to the transparency of the template. However, removal of the quartz component was a significant challenge, even though the nanowire could be identified within the template. Several methods was attempted to achieve the removal of the template, with the conclusion that the quartz component would be best removed using polishing and FIB processing. The process used to fabricate the electrical contact on the bismuth nanowire is summarized in Figure 1. Firstly, a side of the quartz template was removed by polishing, as shown in Figure 1 (a). The typical diameter of the template was over 0.5 mm, which made the polishing process relatively easy using an optical microscope. Polishing was continued to achieve a distance of *ca.* 1 μm between the template surface and the bismuth nanowire. The roughness of the polished side surface was less than ± 50 nm. A thin film Pt-Pd layer (*ca.* 10 nm) was deposited on the polished surface to prevent charge up during the FIB process. The layer was completely removed by FIB processing after the fabrication of the electrodes was completed. The bismuth nanowire sample was installed into a dual-beam FIB apparatus capable of scanning ion microscopy (SIM) and scanning electron microscopy (SEM). The typical vacuum used for FIB processing was approximately 4.0 × 10^−5^ Pa. During FIB processing, the position of the nanowire was not detected by SIM or SEM; however, the approximate position along the wire length was visualized using a position coordinate of the microscope. Gallium (Ga) ion beam sputtering was employed for FIB processing, with detection of the bismuth nanowire position, as shown in Figure 1 (b). The typical area of removed template was 3 × 10 μm, corresponding to the wire length direction and its perpendicular, respectively (Figure 2a). A very slow sputtering rate was used for careful identification of the wire from SIM imaging. The SIM imaging could not directly detect the bismuth nanowire signal unless the wire was exposed using FIB processing. However, the use of an accelerated Ga ion beam (30 kV) made it possible to detect where the nanowire was lying, even if the wire was not completely exposed, and this was recognized under low vacuum SEM with application of a high voltage. Unfortunately, the acceleration voltage and angle of the SEM detector in the dual-beam FIB apparatus were not suitable to identify the wire position when covered with quartz. The high voltage energy Ga ion beam could penetrate the low density quartz template and could be detected at the bismuth nanowire surface. Therefore, the density difference was detected by SIM imaging, as shown in Figure 2a. The FIB processing was stopped right after the bismuth nanowire could be detected. The thickness between the surface of the template and the nanowire was estimated to be less than 500 nm. After successfully determining the position of the bismuth nanowire, rectangular portions of the template adjacent to the nanowire were removed by FIB to a depth of 5 μm from the quartz surface and a distance of 650 nm from the edge of bismuth nanowire (Figures 1 (c) and 2b). The template with bismuth nanowire was then tilted (*ca.* 60º) to expose each wire side for FIB processing, as shown in Figure 1 (d). A small area of the quartz wall (typically 1 × 1 μm) was exposed to the Ga ion beam (Figure 2c) and was gradually eliminated under low current. The structure was not observed even if the nanowire was located in there because the nanowire was packed into the quartz template. The FIB was stopped when any structure was identified, as shown in Figures 1 (e) and 2c. Figure 3 shows an SEM image of the exposed side of the wire in the quartz template after completion of the FIB processing. The inset of Figure 3 shows a magnified image after FIB processing, which shows the diameter of the FIB hole was less than 100 nm. Therefore, there is a possibility to apply this technique to nanowires with diameters less than 100 nm. The detected position of the bismuth nanowire was consistent with that expected in Figure 2a and with the estimated distance between the template surface and nanowire. Therefore, we could conclude that this is a bismuth nanowire in the quartz template. Further processing was required at the opposite side (Figure 2d) to achieve a gap between both sides of the wire of typically <1 μm size, to enable taking Hall measurements with very small error. Inset of Figure 2d shows optical microscope image of the processed area. The nanowire could be observed through the quartz template. Thereby removal of the quartz component was successfully achieved using the FIB process without oxidation of the nanowire surface.

## Results and discussion

**Figure 4:**
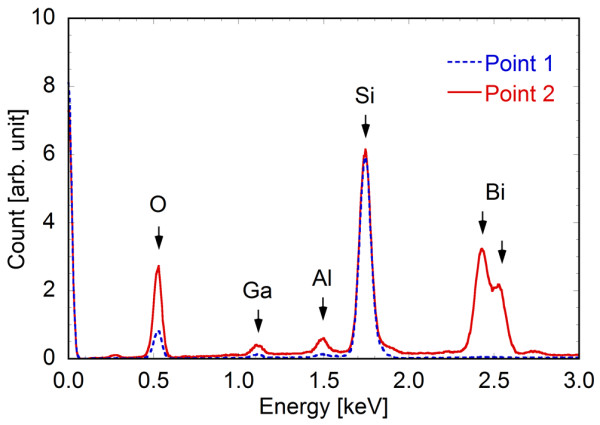
**EDX spectra at points 1 and 2, as indicated in Figure**3.

It should be noted that there is a possibility that the bismuth component is vaporized when exposed to the Ga ion beam. Therefore, energy dispersive X-ray (EDX) analysis was performed at points 1 and 2 shown in Figure 3. Point 1 is the FIB-processed quartz template and is composed of Si and O as the main components of quartz, as shown in Figure 4. Although a Ga component was also observed due to the Ga ion beam processing, no bismuth component was detected at point 1. In contrast, at point 2 which was located in the center of the exposure area for the nanowire, the bismuth component was successfully detected (Figure 4). The electron beam for EDX has a typical spread of 1 × 1 μm; therefore, Si and O components were also detected at point 2. Al was also detected as a component of the microscope stage. The difference between points 1 and 2 indicates that the bismuth component remained after FIB processing.

**Figure 5:**
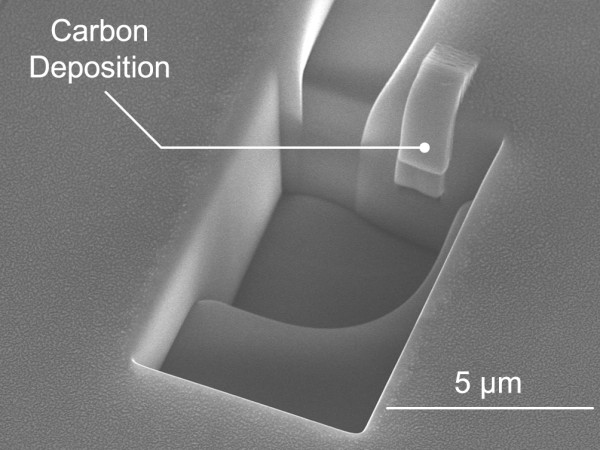
SEM image after carbon deposition onto the exposed side surface of the bismuth nanowire.

Carbon electrodes were then deposited *in situ* on the exposed wire side surfaces to avoid oxidation and provide electrical contact points. Figure 5 shows a typical SEM image of carbon deposition on the exposed nanowire surface. The typical thickness of the carbon deposition was *ca.* 0.5 μm. After achieving the electrical contact, the carbon film layer was then extended to a copper electrode on the surface of the quartz template, as shown in Figure 1 (f) and (g). The expected length of carbon between the surface of the bismuth nanowire and copper electrode is approximately 20 μm; therefore, the resistance of the carbon component was estimated to be *ca.* 200 Ω at an assumed resistivity of 50 μΩ m. The resistance would be reduced for a thicker carbon film.

**Figure 6:**
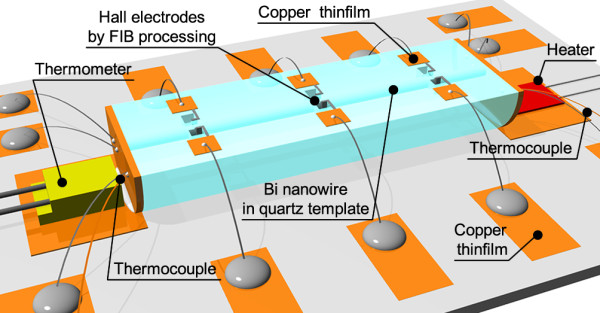
Schematic diagram showing the configuration used for Hall measurement of the bismuth nanowire.

Figure 6 shows a schematic diagram of the configuration used for Hall measurements after polishing, FIB processing, and carbon deposition of the nanowire. The bismuth nanowire is fixed on an alumina plate as a back base prepared with copper electrodes. A thermometer is attached on the plate to measure the temperature. The thermocouple and heater are also attached in order to measure thermoelectromotive force for measurement of a Seebeck coefficient. The lead wires from the pad on the alumina plate are connected to the copper electrode located on the surface of the quartz template. The Hall effect signal in a magnetic field is measured using this configuration, which also makes it possible to perform resistance measurements by the four-probe method. The magnitude of the resistance using the two-probe method was relatively large due to the small diameter and long length of the nanowire. For example, the resistance of a bismuth nanowire with a diameter of 100 nm and a length of 1 mm was estimated to be *ca.* 166 kΩ for an assumed resistivity of 1.3 μΩm. The value is relatively large for the resistance measurements; therefore, the time constant of the circuit should be considered. If the four-probe method is used, then the resistance value can be easily controlled using a suitable gap for measurement of the voltage. Therefore, the configuration shown in Figure 6 enables the successful measurement of the electrical properties of nanowires, including not only the resistivity and Hall coefficient, but also the Seebeck and Nernst coefficients.

The magnitudes of the Hall voltage measured for the nanowire are now discussed. The Hall coefficient (*R*_H_) for a two-carrier model is expressed by $R_{H}=\frac{r_{H}}{e}\frac{p-nb^{2}}{{\left(p+nb\right)}^{2}}$$b=\frac{\mu_{n}}{\mu_{p}}$ , where *r*_H_ is the Hall factor, *n* and *p* are the electron and hole carrier densities, respectively, and *b* is the mobility ratio between electrons μ_*n*_ and holes μ_*p*_[32]. The value of *r*_H_ is almost 1, even if carrier scattering processes are assumed. The values of *n* and *p* are taken from [7] for that of bulk Bi. The *b* value was estimated to be from 3 to 10, depending on the crystal orientation [33], and a value of *b* = 3 was selected as a severe assumption. The Hall resistance *R*_Hall_ is approximately expressed as $R_{\text{Hall}}=R_{H}\frac{Bl}{S}=\frac{4R_{H}B}{\pi d}$, where *d* and *B* are the wire diameter and the magnitude of the magnetic field, respectively. Here the cross section of the nanowire (*S*) and the gap between metal electrodes for the Hall measurement (*l*) are approximated by $S=\pi {\left(\frac{d}{2}\right)}^{2}$ and l=d, respectively. Conditions of *d* = 100 to 1,000 nm were considered in *B* = 0.12 T, which satisfy the low magnetic field approximation at 300 K [34]. Therefore, the calculated |*R*_Hall_| is estimated to be from 0.19 Ω at 1,000 nm to 1.9 Ω at 100 nm. The electric current introduced into the bismuth nanowire for the measurement should ideally be less than 1 μA due to the small heat capacity of the narrow wire and the high resistance, so that an increase in temperature and burn out can be avoided. The absolute value of the measured Hall voltage was estimated to be 190 nV to 1.9 μV under the same conditions. Nanovolt-order voltage can be measured using a lock-in amplifier with modulation of the introduced current and magnetic field, which confirms that the configuration (Figure 6) enables the experimental measurement of the Hall coefficient. The Hall coefficient of the bismuth nanowire enables us to evaluate carrier density and mobility experimentally. Therefore, the temperature dependence of resistivity of bismuth nanowires less than 1 μm can be described by the measurement of carrier density and mobility more directly than the calculation model of our previous study. In ition, it will be possible to measure not only Hall coefficient but also Nernst coefficient using the configuration; then, various electrical properties of bismuth nanowires can be evaluated.

## Conclusions

A method to attach Hall electrodes on opposite sides of an individual bismuth nanowire encased in a quartz template was demonstrated using polishing, FIB processing, and carbon deposition. An accelerated Ga ion beam was used to detect the position of the nanowire in the quartz template without exposure. The quartz component covering the nanowire was then successfully removed with the Ga ion beam to locally expose the opposite side surfaces of the nanowire. Carbon thin films were then deposited *in situ* on the exposed wire surfaces to form electrical contacts, while avoiding oxidation of the nanowire. EDX analysis of the processed area indicated that the bismuth component remained after FIB processing.

The mobility reduction of bismuth nanowires less than 1 μm in diameter due to mean free path limitation at wire boundary will be evaluated experimentally by Hall measurement. Moreover, further investigations are planned using the configuration to examine the temperature and wire diameter dependence of the thermoelectric properties of bismuth nanowires, and the influence of the quantum effect using much smaller diameter of bismuth nanowires will be also estimated.

## Competing interests

The authors declare that they have no competing interests.

## Authors' contributions

MM carried out experiments, the polishing, FIB processing, EDX analysis, and drafted the manuscript. YH and TK guided the idea and the experiments and revised the manuscript. TK participated in the design of the FIB processing. All authors read and approved final manuscript.

## Authors' information

MM is a PhD candidate under associate professor YH in the department of engineering, Saitama University, Japan.
